# Urgent action to fight hepatitis C in people who inject drugs in Europe

**DOI:** 10.1186/s41124-016-0011-y

**Published:** 2016-06-30

**Authors:** John F. Dillon, Jeffrey V. Lazarus, Homie A. Razavi

**Affiliations:** 1 Division of Molecular and Clinical Medicine, School of Medicine, University of Dundee, Ninewells Hospital, Dundee, UK; 2 Centre for Health and Infectious Disease Research (CHIP) and WHO Collaborating Centre on HIV and Viral Hepatitis, Rigshospitalet, University of Copenhagen, Copenhagen, Denmark; 3 Center for Disease Analysis, Louisville, Colorado USA

**Keywords:** Antiviral therapy, Harm reduction, Hepatitis C, People who inject drugs, Risk reduction behaviour

## Abstract

Hepatitis C virus (HCV) infection is a leading cause of liver cirrhosis and liver cancer, is curable in most people. Injecting drug use currently accounts for 80 % of new HCV infections with a known transmission route in the European Union (EU). HCV has generally received little attention from the public or policymakers in the EU, with major gaps in national-level strategies, action plans, guidelines and the evidence base. Specifically, people who inject drugs (PWID) are often excluded from treatment owing to various patient, healthcare provider and health system factors.

All policymakers responsible for health services in EU countries should ensure that prevention, treatment, care and support interventions addressing HCV in PWID are developed and implemented. According to current best practice, PWID should have access to comprehensive, evidence-based multiprofessional harm reduction (especially opioid substitution therapy and clean needles and syringes) and support/care services based in the community and modified with community involvement to accommodate this hard-to-reach population. Other recommended components of care include vaccination against hepatitis B and other infections; peer support interventions; HIV testing, prevention and treatment; drug and alcohol services; psychological care as needed; and social support services. HCV testing should be performed regularly in PWID to identify infected persons and engage them in care. HCV-infected PWID should be considered for antiviral treatment (based on an individualised assessment and delivered within multidisciplinary care/support programmes) both to cure infected individuals and prevent onward transmission. Modelling data suggest that the HCV disease burden can only be cut substantially if antiviral treatment is scaled up together with prevention programmes. Measures should be taken to reduce stigma and discrimination against PWID at the provider and institutional levels.

In conclusion, strategic action at the policy level is urgently needed to increase access to HCV prevention, testing and treatment among PWID, the group at highest risk of HCV infection. Such action has the potential to substantially reduce the number of infected persons, along with the disease burden and related care costs.

## Background

According to the World Health Organization (WHO), 14 million people in the European Region are chronically infected with hepatitis C virus (HCV), a blood-borne infection of the liver [1]. Another recent study has estimated that there are 3.6 million people with viraemic HCV infections in the EU [2]. HCV is a leading cause of liver cirrhosis and liver cancer and around 70,500 infected people die each year in the European Union as a result of these complications [3]. Without action, the burden of HCV disease is expected to rise in the coming years owing to the ageing of the infected population, ongoing new infections and low levels of treatment uptake [4–6]. For example, the number of HCV-related cirrhosis cases in England is expected to increase by 50 % by 2030, while HCV-related deaths will almost double [4].

Injecting drug use currently accounts for 80 % of new HCV infections in the European Union with a known transmission route [7]. Globally, two out of three people who inject drugs (PWID) have HCV infection [8], with rates in European Union countries varying between 14 % and 84 % [9]. However, HCV remains a hidden epidemic. A pooled analysis of data from studies in Denmark, France, Poland, Spain and the United Kingdom estimated that a median of 49 % of HCV-infected PWID were undiagnosed (range: 24–76 %) [10]. HCV is transmitted more easily than HIV and globally there are more than three times as many PWID infected with HCV than with HIV [8]. When formulating strategies to address the problem of HCV infection, especially if considering treatment as prevention strategies, it is important to differentiate between individuals who are currently active drug injectors and those who are former injectors. In addition, among active injecting drug users, it is important to distinguish between those who share injecting equipment and those who do not. HCV is spread between PWID primarily by the sharing of injection equipment and thus the vast majority of new infections occurs in active injectors who share.

The window of opportunity for HCV prevention is narrow, as infection is likely to occur soon after initiation of injection drug use. The time to infection is shortest among individuals with many contacts within their network of drug users and among those who inject most frequently [11]. Furthermore, the incidence of infection is highest among individuals who do not utilise existing harm reduction services such as opioid substitution therapy (OST) and sterile needle and syringe programmes (NSP). HCV in PWID is preventable and curable, and therefore efforts to reduce the incidence and transmission of HCV infection must aim to increase access to testing, risk reduction and treatment in this population, with a strong focus on active injectors. Treatment with antiviral medicines is as effective in PWID as in non-drug users [12], and guidelines published by international and European organisations identify the prevention and treatment of HCV among PWID as major components of protecting the public from blood-borne viruses [13–16].

The European Commission recognises PWID as a key target group in its ongoing activities to address blood-borne viruses, and in October 2015 it launched a three-year Joint Action that engaged representatives of 18 Member States in efforts to intensify the response to HIV and viral hepatitis among PWID [17] and followed this up with a second Joint Action on these issues in 2016. However, considering the extent of the public health threat, HCV has generally received little attention from the public or policymakers in the European Union, with major gaps in international and national-level strategies, action plans, guidelines and in the evidence base more generally [10, 18–20]. This paper discusses barriers to improving HCV care for PWID and current best practices in HCV prevention, diagnosis and treatment in PWID in the context of recent data and relevant policy initiatives. It concludes by making policy recommendations to address unmet needs in PWID and thereby strengthen the response to HCV in the European Union.

### Barriers to improving HCV care for PWID

A recent study of the EU countries found an overall diagnosis rate for HCV infection of 33 % and a treatment rate of 3.7 % [2]. Median treatment rates specifically among diagnosed PWID have been estimated at around 10–30 % [10, 21], but this masks wide variation within and between countries and of course many PWID are not even diagnosed. A study of seven sites in the UK found that treatment rates among PWID varied from <5 to >25 per 1000 PWID [22]. Of course, many more PWID are not even diagnosed. Access to prevention, testing and treatment is even more limited in prisons than in the community, despite high rates of HCV in the prison population internationally [23, 24] as well as legal and human rights obligations for governments to provide health care to prison inmates.

A fundamental dilemma is that PWID, in whom HCV infection is most common and in whom treatment to prevent onward transmission is particularly important, are generally difficult to engage in formal healthcare services. PWID are often wrongly excluded from treatment, and uptake rates are low even where treatment is offered. The main barriers to PWID accessing care for HCV include [21, 25–28]:

- *Patient-related factors*, such as lack of awareness of diagnosis or HCV status; limited knowledge or negative perceptions of HCV and how it is treated; low levels of health literacy; negative relations with the healthcare system, including fear of or experience of stigmatization; social, medical and psychiatric co-morbidities; lack of insurance; low socioeconomic status; homelessness or unstable housing; and factors relating to migrant status or ethnic/cultural minority status;
- *Healthcare provider-related factors*, such as limited expertise in HCV care for PWID; misperceptions that treatment is less effective or is associated with high re-infection rates in PWID versus non-PWID groups; and discrimination or stigmatization toward PWID;
- *System-related and institutional factors*, such as lack of infrastructure or treatment settings adapted or conveniently located for PWID; lack of coordination or collaboration among providers of different services; lack of suitable training programmes; high treatment fees; criminalisation of PWID; and a lack of validated, shared, systematic national data on the health and economic impact of HCV to inform service investment and planning.

Programmes for HCV treatment as prevention will only work if high levels of coverage and adherence are achieved and maintained. In light of the known barriers, HCV services need to be modified to effectively deliver harm reduction interventions and antiviral treatment to hard-to-reach populations. Survey data suggest that the majority of PWID may be willing to receive treatment for HCV if it is available to them [29]. However, whether individuals undertake and adhere to treatment in practice will depend on how they prioritise this option in relation to various competing priorities and challenges [26]. Some individuals may need particular encouragement, making advocacy and peer support important features of efforts to scale up treatment.

The question of whether further measures to incentivise PWID into care should be employed, and what form these should take, has received little attention to date. In conventional health services, patients with symptoms are motivated to seek health care as a means of obtaining relief from the symptoms. Similarly, patients are motivated to participate in HCV testing programmes because of their recognition that a diagnosis can lead to the timely initiation of treatment, reducing the risk of complications from the disease. In both cases, health-seeking behaviour is rewarded. In the PWID population, these mechanisms may be much less powerful. PWID often have quite nihilistic views of the future [30, 31], and as HCV is frequently asymptomatic, there are few drivers for treatment-seeking behaviour. This is compounded further if early treatment is perceived to principally benefit society (in terms of prevention of transmission, as discussed below) rather than infected individuals. Such perceptions may result from the asymptomatic nature of early infection or a lack of knowledge or concern about the long-term consequences of chronic HCV infection among some PWID. Additionally, for treatment as prevention strategies to work, the uptake of treatment needs to be high, as a small number of individuals at key points in networks could reinfect many people who have been cured. Therefore, incentives or contingency management programs should be considered. Assessed in a variety of diseases and populations by the National Institute for Health and Care Excellence in England and Wales and found to be cost-effective and acceptable [32], these should be considered a key component of motivating active PWID populations into treatment.

Crucially, many countries lack national evidence-based best practice programmes such as the one implemented and evaluated in Scotland [33]. In 2006, the Scottish Government launched its Hepatitis C Action Plan for the purposes of improving services to prevent transmission of HCV infection (particularly among PWID), identifying those infected, and ensuring that those infected receive optimal treatment, care and support [33]. The comprehensive plan was based on evidence from national monitoring systems and models showing the potential benefit of scaling up therapy and the mounting cost of inaction, and was informed by stakeholder consultations. It was implemented by national and local multidisciplinary, multi-agency networks, coordinated by Health Protection Scotland and supported by substantial government investment. Achievements include an increase of approximately 50 % in the proportion of the infected population diagnosed (38 % to 55 %); a nearly three-fold increase in the annual number of PWID initiating therapy, and a reversal of the upward trend in the overall number of people living with chronic HCV infection [33].

### Prevention, harm reduction and testing

**Fig. 1:**
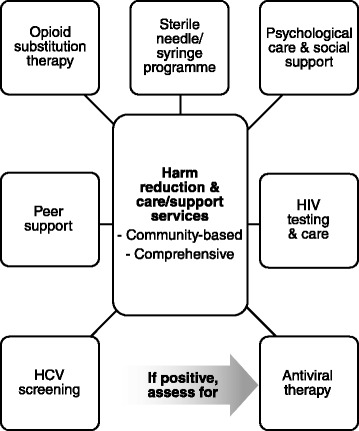
Components of comprehensive hepatitis C virus (HCV) care services to which people who inject drugs should have access

There is currently no vaccine against HCV. However, HCV infection among PWID can be greatly reduced using a combination of prevention strategies [13, 34]. PWID should have access to comprehensive harm reduction and support/care services. These services should also be based in the community, to facilitate access by PWID and to aid service expansion. Guidelines by WHO [13], the Joint United Nations Programme on HIV/AIDS [35], the European Centre for Disease Prevention and Control (ECDC)/European Monitoring Centre for Drugs and Drug Addiction [14] and other experts [15, 16, 36] all recommend packages of services that include the following elements (Fig. 1).

### Prevention and harm reduction

PWID should have easy access to harm reduction services, two key components of which are OST and NSP [13, 14, 35]. As well as reducing HCV risk behaviour and transmission through injecting drug use, these interventions can serve as a platform to promote HCV care. While harm reduction services are often grouped together, it is important to differentiate them and their potential utility in efforts to combat HCV. NSP programmes by definition are focussed on those still injecting and aim to make the act of injecting safer. Such services greatly reduce the rate of transmission. Therefore, it is logical to make this group the focus of treatment-as-prevention strategies. OST services take many forms across countries and regions depending on local legal requirements and regulations; some are very open to unstable and active injectors while others require PWID to modify their drug use to varying degrees as a condition for initiating or continuing OST. The design of the OST service model has considerable implications for the efficacy of OST as a prevention tool for HCV, with the only likely prevention benefit being seen in the context of low-threshold early access leading to reduced injecting frequency. The use of peer support interventions within these services is recommended [13, 14, 35]. Various models of peer support exist [37], including those that promote education, engagement in services, and treatment uptake and assessment [37–40]. HIV testing, prevention and care should be provided in concert. Available data suggest that the prevalence of HIV/HCV co-infection ranges widely across Europe, depending on the prevalence of HIV [10]. Other recommended components of care for PWID include: vaccination against other infections (e.g. hepatitis A and B, tetanus), psychological care for selected cases, and social support services [13–16, 35, 41].

The 2013–2016 European Commission Action Plan on Drugs charges Member States with ensuring that treatment and outreach services incorporate greater access to risk and harm reduction options to reduce the consequences of drug use including transmission of blood-borne viruses [42]. There are examples of good practice underway in Europe [33, 43], but the unmet need is still high [44, 45].

### Testing

Many individuals with chronic HCV infection are diagnosed after being infected for many years and enter care only when they develop clinical symptoms, despite having been engaged by the healthcare system earlier [46]. Late diagnosis of HCV is associated with worse outcomes [46] and with the risk of onward transmission. Many opportunities for timely diagnosis of HCV infection are missed, especially in PWID populations. Active testing of PWID who are current injectors is rarely undertaken. It is argued that treatment of such patients is too difficult, too expensive and the risk of re-infection too high, although the developing evidence base clearly shows that this is not the case. Additionally, the diagnosis of HCV may change behaviour in the short term or identify patients who could be followed up for treatment in the future before presenting with liver failure. Recently, the European Association for the Study of the Liver and the HIV in Europe initiative developed a new consensus definition of late presentation for viral hepatitis [47]. This is an important performance indicator of success for programmes aiming to improve the identification and management of viral hepatitis.

HCV testing should be performed regularly in PWID to identify infected persons and engage them in care and treatment [13–16]. HCV antibody testing is recommended, and if the result is positive, a more sensitive HCV RNA test should be performed to determine whether or not there is current infection [13, 16]. Targeted HCV testing linked to treatment increases diagnosis rates and treatment uptake [48] and is a cost-effective way to reduce the disease burden [49]. New types of rapid and point-of-care HCV tests (e.g. dried blood spot tests) can help to increase the number of tested individuals, especially in hard-to-reach groups [13, 50]. However, according to a 2013 survey, only six of 44 countries in the European Region provided hepatitis B and C virus testing to PWID at no charge [20].

### Antiviral treatment: individual benefit and transmission prevention

HCV antiviral treatment aims to cure, or eradicate, HCV infection in individuals, thereby preventing its complications and death [15]. HCV treatments are assessed according to the sustained virological response rate (SVR), defined as the proportion of patients with undetectable HCV RNA measured after 12 or 24 weeks according to defined methods. Patients who achieve an SVR remain free of the virus in 99 % of cases and hence are considered cured [15, 51]. An SVR has been associated with significantly reduced risks of 10-year all-cause mortality, and liver-related mortality or transplantation, among HCV-infected individuals with advanced hepatic fibrosis [52].

The introduction of direct-acting antiviral (DAA) therapies in recent years has revolutionised the treatment of HCV. Previously, pegylated interferon/ribavirin (peg-IFN/RBV) regimens were only moderately effective (depending on the HCV genotype), required IFN injections and variable treatment durations up to 48 or even 72 weeks, and were associated with significant toxicities. New all-oral IFN-free DAA regimens, given for 8–24 weeks, are much more effective (achieving SVR rates of ≥90 % in clinical trials), and are generally well tolerated [13, 15, 53]. It can be extrapolated that the much higher SVRs achieved will still be associated with the reduced liver and all-cause mortality seen with interferon-based regimens.

Currently, treatment priority is based on fibrosis stage, risk of progression to more advanced disease, presence of extrahepatic manifestations of infection and cirrhosis. It should also take into account the risk of transmission [15]. European and international guidelines recommend that all patients with chronic HCV — including PWID — should be considered for antiviral treatment based on an individualised assessment [13–16, 35]. According to a systematic review of studies, the effectiveness of peg-IFN/RBV treatment among PWID who are eligible and committed to starting HCV treatment (including active injectors) is similar to that in non-drug users, with high levels of adherence, low rates of treatment discontinuation, and a low rate of reinfection among PWID [12]. These findings support current treatment recommendations and refute misperceptions about poor effectiveness and high rates of re-infection in PWID that can lead to their exclusion from treatment. Indeed, PWID are among the groups that should be prioritised for treatment, regardless of fibrosis stage or extra-hepatic manifestations, in view of their risk of transmitting HCV [15]. Patients who are co-infected with HCV and HIV should also be prioritised for HCV treatment [15]. Importantly, HCV treatment for PWID should be delivered within multidisciplinary care/support programmes that include harm reduction and support services [13–16, 35], including measures to limit alcohol intake and to address obesity, smoking and drug usage.

Curing HCV infection not only benefits infected individuals, but also has the potential to prevent onward viral transmission and hence to reduce the disease burden at the population level [13]. The impact of treatment on HCV prevalence depends on its effectiveness and on the levels of treatment uptake. Modelling studies in various countries consistently suggest that current levels of treatment using conventional peg-IFN/RBV regimens are unlikely to significantly reduce or even stabilise the disease burden over the next 10 to 15 years [4–6, 54]. According to models, the HCV disease burden can only be cut substantially if antiviral treatment is scaled up together with prevention programmes [22, 54–60]. For example, using dynamic modelling, researchers in the United Kingdom projected that a 50 % reduction in the prevalence of chronic HCV in PWID (from a baseline of 20 %, 40 % or 60 %) could be achieved within 10 years by combining OST, NSP and antiviral treatment. A halving of the prevalence was made more achievable (i.e. fewer treatments were needed) if DAAs were used instead of peg-IFN/RBV, and also if harm reduction were scaled up [55]. Further modelling, based on real-world data on treatment rates and outcomes from 538 PWID in seven centres in the United Kingdom, suggested that the introduction of DAA therapies combined with the scaling up of treatment to rates already achieved in some centres – to 26 per 1000 PWID – could allow all seven sites to achieve at least a 15 % absolute reduction in HCV prevalence after 10 years, with prevalence more than halved in three sites [22]. In Scotland, doubling treatment uptake and prioritising PWID was projected to reduce incident infection to negligible levels (<50 cases per year) by 2025 and to stabilise rates of severe liver morbidity by 2028 [61]. Targeting those with moderate/advanced fibrosis would stabilise severe liver morbidity five years earlier, but would be significantly less effective in reducing new infections. In Sweden, modelling suggests that doubling the number of DAA treatments overall would bring about 65–70 % reductions in both the incidence of hepatocellular cancer and liver-related deaths by 2030 [59]. Targeting only patients with advanced fibrosis would only stabilise the incidence of serious complications with minimal impact on the total number of HCV infections. Internationally, other analyses suggest that a 90 % reduction in total HCV infections and/or disease burden within 15 years is feasible in most countries studied, provided there is upscaling of testing, harm reduction and efficacious treatment [5, 57].

Antiviral treatment among PWID, in combination with harm reduction services, is therefore a critical component of primary prevention of HCV. Despite implementation challenges, treatment uptake rates of 20–22 % have already been reported in dedicated services linking OST and community hepatitis care [22, 62–64]. Furthermore, experience from Scotland shows that a scale-up of antiviral therapy can be achieved without compromising SVR rates, including among PWIDs [65].

The cost of DAA treatments is a barrier to their wide usage in HCV management strategies in most European Union countries. However, PWID were not widely treated even during the previous era of lower cost therapies, underlining the point that particular barriers such as discrimination exist for this group. Economic models indicate that HCV treatment using DAAs is cost-effective owing to a reduction in the costs of HCV-related complications [66–69]. Few studies have assessed the cost-effectiveness of antiviral treatment specifically for PWID (Table 1). Modelling data from Australia and the United Kingdom suggest that treating active or former PWID with peg-IFN/RBV at mild stages of HCV is cost-effective compared with no antiviral treatment, resulting in incremental cost-effectiveness ratios (ICER, measured as cost per quality adjusted life-years [QALY]) below standard local thresholds for cost-effectiveness [70, 71]. The Australian study and the UK study both took into account the costs of disease progression, together with deleterious aspects such as reinfection, but only one also took into account the prevention effects of treatment on transmission [70].

**Table 1:** Studies modelling the cost-effectiveness of antiviral therapy for hepatitis C virus infection in people who inject drugs (PWID)

Reference	Country/setting	Design	Intervention and population	Cost impact
PegIFN/RBV therapy				
Martin et al. 2012 [70]	United Kingdom	Dynamic disease progression and transmission model	pegIFN/RBV at mild stage vs no treatment (best supportive care) in:	ICER for treating current PWID vs no treatment, according to baseline chronic HCV prevalence:
		Probablistic cost-utility analysis	Current PWID	20 % prevalence: ICER treat PWID vs no treatment = £521/QALY
		Direct medical costs (2010 prices)	Non/ex PWID	40 % prevalence: ICER vs no treatment = £2539/QALY
			N = 1000 individuals	60 % prevalence: ICER = £7675^a^/QALY
				Treatment of non/ex-PWID dominant at 60 % prevalence; ICER £6803/QALY vs no treatment
Visconti et al. 2013 [71]	Australia	Markov decision-analytic model	pegIFN/RBV at mild (F0/1) stage vs no treatment (best supportive care) in:	Current PWID: $AUS 7941/QALY
		Direct medical costs (2011 prices)	Current PWID	Former PWID: $AUS 5808/QALY
			Former PWID	Non-injectors: $AUS 3985/QALY
			Non-injectors	Treatment at mild stage dominated treatment at later stages for all cohorts
			N = 1000 individuals	
DAA therapy				
Bennett et al. 2015 [58]	United Kingdom	Dynamic model of disease progression, transmission and treatment	Uptake increased to 250 per 1000 PWID of:	2015–2027
			Current treatment	Current treatment: £23.4 million saved (£5.4 after discounting)
			New DAA (SVR90%)	SVR90%: £36.3 million saved (£8.4 million after discounting)
		Lifetime complication rates, costs of complications	N = 4240 individuals	
Hellard et al. 2015 [72]	Australia	Closed compartmental model of disease progression and treatment	IFN-free DAA at	Late treatment vs no treatment: $AUS5078
			Early stage (from F0)	Early treatment vs late treatment: $AUS17,090
		Fixed rate of re-infection	Late-stage (from F2/3)	
		Direct healthcare costs (2014 prices)	N = 1000 individuals	
Scott et al. 2016 [73]	Australia	Open compartmental model of progression, transmission and treatment	DAA treatment scale up necessary to achieve WHO goals of 65 % reduction in HCV-related deaths and 80 % reduction in HCV incidence by 2030 via two scenarios if DAA treatment for IDU-acquired HCV prioritised to: Patients with advanced liver disease (F ≥ 3) or Current PWID	Prioritising advanced liver disease: Mortality target required 5662 (95 % CI 5202–6901) courses/year (30/1000 IDU-acquired infections)
				Prioritising PWID:
				Incidence and mortality targets achieved with 4725 (95 % CI 3278–8420) courses per year (59/1000 PWID)
				Additional 5564 (1959–6917) treatments/year (30/1000 IDU-acquired infections) required for 5 years for patients with advanced liver disease to avoid excess HCV-related deaths
				ICER: $AUS25,121 ($AUS11,062–$AUS39,036)/QALY

*DAA* direct acting agents, *F0–3* METAVIR score, *ICER* incremental cost-effectiveness ratio, *pegIFN/RBV* peglyated interferon/ribavirin, *QALY* quality-adjusted life-years, *SVR* sustained virological response

^a^Calculated from data in published source

Recently, at least three further modelling studies have evaluated the impact and cost-effectiveness of interferon-free DAAs in PWID. One study, based on UK data and integrating disease progression and transmission according to a previous model [55], projected that an increase in uptake of conventional treatment with a 58 % SVR from 10 per 1000 PWID to 250 per 1000 PWID (among a modelled population of 4240 PWID with HCV prevalence of 25 %) would result in cost savings of £5.4 million between 2015 and 2027 after discounting at 3.5 % per annum [58]. Using newer treatments with a 90 % SVR was projected to increase the amount saved to £36.3 million, or £8.4 million after discounting [58].

In Australia, Hellard et al. modelled the expected healthcare costs and QALYs among newly HCV-infected PWID according to three scenarios: no treatment; ‘early’ treatment after initial infection; or ‘late’ treatment prior to developing compensated cirrhosis [72]. Compared with no treatment, early treatment and late treatment were associated with ICERs of AUS $10,272/QALY (95 % CI $5689–13,690) and AUS $5078/QALY (95 % confidence interval $2847–5295), respectively. Both of these values are well below the unofficial Australian willingness to pay threshold of AUS $50,000/QALY. Early treatment was the most effective option in terms of QALYs gained, while late treatment was associated with a lower ICER because of a lower likelihood of re-infection in patients treated later in the course of infection due to the cessation of injection drug use. These researchers acknowledged that their analysis may have underestimated the cost-effectiveness of treatment for two reasons: it did not take into account any benefits from reduced infection transmission, and the costs of care for cirrhosis and liver cancer were based on minimum requirements [72].

Most recently, these authors have used a model that includes HCV transmission as well as disease progression and treatment, to estimate the treatment scale up and cost-effectiveness of reaching WHO targets of an 80 % reduction in incidence and 65 % reduction in HCV-related deaths by 2030, specifically among PWID [73]. According to this analysis, achieving the mortality target would require treatment to be scaled up to 30/1000 injecting drug use-acquired infections per year among patients with advanced liver disease, while the incidence target required 59/1000 PWID to be treated each year. Prioritising treatment at this level only to PWID would achieve the mortality target with fewer treatment courses in total, but at the expense of a clinically unacceptable number of deaths among individuals with advanced disease. Hence, additional treatments (at the aforementioned rate, 30/1000/year) were required for patients with advanced liver disease for the first five years. Achieving both targets in this way was associated with an ICER of AUS $25 121 (AUS$11 062–AUS$39 036) per QALY gained, below the unofficial cost-effectiveness threshold [73].

## Conclusion and recommendations

The hepatitis C pandemic is a pressing public health threat. Strategic action at the policy level is urgently needed to increase access to HCV prevention, testing and treatment among PWID, the group at highest risk of HCV infection. Such action has the potential to substantially reduce the number of infected persons, along with the disease burden.

The World Health Organization has called for a global movement to create generalized access to HCV treatment [13], as have civil society organisations and other stakeholders [74]. They have further called for national and European strategies on HCV that include specific measures for PWID [28, 75, 76]. In 2014, the 67th World Health Assembly approved a resolution urging Member States to develop and implement coordinated multi-sectoral national strategies for preventing, diagnosing and treating viral hepatitis based on the local epidemiological context [77]. The first-ever WHO Global Health Sector Strategy on Viral Hepatitis (2016–2021) was adopted by the 69^th^ World Health Assembly on 28 May 2016 [78]. Aiming to contribute to the United Nations Sustainable Development Goals [79] and to eliminate viral hepatitis as a public health threat by 2030, the strategy highlights the opportunities available through investing in an essential, priority set of core interventions that include comprehensive integrated harm reduction services for PWID, linked with treatment [78]. This global strategy is complemented by a draft WHO European Region action plan for consideration by the Regional Committee in September 2016 [80]. Additionally, in the 2014–2016 EU Action Plan on HIV and Co-infections, the European Union has committed to work with Member States, neighbouring countries, civil society and the ECDC to implement risk and harm reduction measures for PWID and their partners for the prevention and treatment of HIV, co-infections and drug dependency in the community and prisons [81].

All policymakers responsible for health services in European Union countries should ensure that:

- National HCV action plans and strategies that include measures specifically to address HCV in PWID are developed and implemented across Europe;
- Evidence-based, comprehensive, multi-professional, community-led harm reduction and care/support programmes are provided and linked to HCV testing and treatment in the public and prison health systems;
- PWID have equitable access to effective HCV treatment, in line with published guidelines;
- Measures are undertaken to reduce stigma and discrimination against PWID at the provider and institutional levels;
- HCV awareness and prevention campaigns are undertaken for healthcare providers (doctors, nurses and allied health professionals) and the PWID community; and
- The PWID community is involved in HCV service planning and implementation, with consideration given to peer support and PWID ‘champions’ initiatives.

## Abbreviations

DAA, direct-acting antivirals; ECDC, European Centre for Disease Prevention and Control; HCV, hepatitis C virus; ICER, incremental cost-effectiveness ratio; NSP, needle and syringe programme; OST, opioid substitution therapy; Peg-IFN/RBV, pegylated interferon/ribavirin; PWID, people who inject drugs; QALY, quality adjusted life-years; SVR, sustained virological response; WHO, World Health Organization.
